# Biomimetic Membranes for Multi-Redox Center Proteins

**DOI:** 10.3390/ijms17030330

**Published:** 2016-03-03

**Authors:** Renate L. C. Naumann, Andreas F. Geiss, Christoph Steininger, Wolfgang Knoll

**Affiliations:** Austrian Institute of Technology GmbH, AIT, Donau-City-Str. 1, 1220 Vienna, Austria; andreas.geiss.fl@ait.ac.at (A.F.G.); christoph.steininger.fl@ait.ac.at (C.S.); Wolfgang.Knoll@ait.ac.at (W.K.)

**Keywords:** multi-redox center proteins, protein-tethered bilayer lipid membrane, cytochrome c oxidase, photosynthetic reaction centers, surface-enhanced infrared absorption spectroscopy, surface-enhanced resonance Raman spectroscopy, laser-scanning confocal microscopy, UV/Vis spectroscopy, his-tag technology, proteo-lipobeads

## Abstract

His-tag technology was applied for biosensing purposes involving multi-redox center proteins (MRPs). An overview is presented on various surfaces ranging from flat to spherical and modified with linker molecules with nitrile-tri-acetic acid (NTA) terminal groups to bind his-tagged proteins in a strict orientation. The bound proteins are submitted to *in situ* dialysis in the presence of lipid micelles to form a so-called protein-tethered bilayer lipid membrane (ptBLM). MRPs, such as the cytochrome c oxidase (C*c*O) from *R. sphaeroides* and *P. denitrificans*, as well as photosynthetic reactions centers (RCs) from *R. sphaeroides*, were thus investigated. Electrochemical and surface-sensitive optical techniques, such as surface plasmon resonance, surface plasmon-enhanced fluorescence, surface-enhanced infrared absorption spectroscopy (SEIRAS) and surface-enhanced resonance Raman spectroscopy (SERRS), were employed in the case of the ptBLM structure on flat surfaces. Spherical particles ranging from µm size agarose gel beads to nm size nanoparticles modified in a similar fashion were called proteo-lipobeads (PLBs). The particles were investigated by laser-scanning confocal fluorescence microscopy (LSM) and UV/Vis spectroscopy. Electron and proton transfer through the proteins were demonstrated to take place, which was strongly affected by the membrane potential. MRPs can thus be used for biosensing purposes under quasi-physiological conditions.

## 1. Introduction

Biosensing based on multi-redox center proteins (MRP) has often been performed on solubilized proteins. The most prominent example of this approach is protein film voltammetry [1,2]. Important information about the electrochemical properties of proteins has thus been obtained. The methods of preparation were mostly the immobilization of micelles. Similar methods have often been applied for use with FTIR spectroscopy. Proteins were immobilized on the surface of an ATR crystal via detergent depletion, drying and rehydration [3]. A large body of valuable information has been assembled, but uncertainties remain as to whether the proteins maintain their full functionality under these conditions. Therefore, designing biomimetic membrane systems on surfaces has been attempted, which would be suitable to entrap MRPs into a functionally active bilayer lipid membrane (BLM) [4,5].

The oriented immobilization of MRPs on surfaces modified with nitrile-tri-acetic acid (NTA) via his-tag technology has proved to be very auspicious in this context, particularly if the immobilization was followed by *in situ* dialysis in the presence of lipid micelles. The proteins are thereby incorporated into a BLM to form what was called a protein-tethered bilayer lipid membrane (ptBLM) [6,7]. The system was later employed by other groups to investigate MRPs such as membrane photoreceptors and complex I from *E. coli* [8,9], whereas we concentrated on the cytochrome *c* oxidase (C*c*O) from *R. sphaeroides.* The protein was genetically engineered with his-tags attached to subunit II. In this configuration, the first electron acceptor Cu_A_ points toward the electrode so that the enzyme can be activated by direct electron transfer (ET) [10]. The C*c*O was investigated via fast scan voltammetry and electrochemical titration followed by surface-enhanced infrared absorption spectroscopy (SEIRAS) and surface-enhanced resonance Raman spectroscopy (SERRS) [11,12,13]. The enzyme could be shown to undergo conformational changes through the uptake of four electrons and to translocate protons across the membrane [14]. These results prompted us to conduct time-resolved tr-SEIRAS on this and other proteins, such as the reaction centers (RCs) and the *bc*_1_ complex of photosynthetic bacteria.

Moreover, we made attempts to expand the range of methodologies applicable to MRPs incorporated in lipid membranes from vibrational to fluorescence and UV/Vis spectroscopies. In this context, we exchanged flat surfaces *versus* particles from µm sized gel beads down to nanoparticles. The concept of the ptBLM was thus shown to be applicable also to particle surfaces.

Hence we have demonstrated that the concept of the ptBLM offers the advantage of the immobilization of MRPs in a strict orientation on various surfaces, an important feature if it comes to the investigation of transport parameters. Hence a particular MRP can be induced to transport ions and/or electrons outside-in or inside-out of the BLM, provided the MRP is available with his-tags attached to either side of the protein. This was available to us in the case of the C*c*O and the photosynthetic reaction centers from *R. sphaeroides* and *P. denitrificans*, respectively. Pros and cons of the different approaches are discussed.

## 2. Results and Discussion

### 2.1. Multi-Redox Center Proteins Immobilized on Planar Surfaces

The most advanced techniques applicable to proteins on planar surfaces are surface-enhanced IR and Raman spectroscopies. The surfaces are enhanced by nano-structures such as nanoparticles (NPs) attached to the plane surface that modulate the electromagnetic field around them. The enhancement comes about by the excitation of localized surface plasmon resonances (LSPR), which are electromagnetic waves that enhance the electromagnetic (EM) field near the metallic nanostructures [15,16]. This EM field rapidly decreases into the liquid phase so that only molecules a few nm away from the metallic surface are affected. A review on surface-enhanced Raman spectroscopy for biomedical diagnostics and imaging can be found in ref. [17]. The theory of SEIRAS and SERRS comprises the high sensitivity of bands whose components are located at a short distance from the surface but also the surface selection rule, which predicts that adsorbed molecules are reflected the stronger the more the transition dipole moment is oriented perpendicular to the surface [16,18]. This means that a component may be presented only in one orientation whereas other orientations are not represented. We had previously developed two-layer gold and silver surfaces with NPs generated *in situ* that were suitable to apply not only electrochemical techniques but also SEIRAS and SERRS to our ptBLM structures. C*c*O incorporated into a ptBLM had thus been investigated by fast scan voltammetry and electrochemical titration followed SEIRAS as well as SERRS [11,12,13]. The same surface was then employed to perform time-resolved tr-SEIRAS [19].

#### 2.1.1. Time Resolved tr-SEIRAS of Cytochrome *c* Oxidase (C*c*O) from *R. sphaeroides*

C*c*O with a his-tag engineered to the C terminus of subunit II was immobilized on the surface of the silicon ATR crystal of an IR spectrometer, covered with a structured gold film. On top of the ATR crystal, a flow cell was mounted and filled with a buffer solution appropriate for the immobilization and subsequent reconstitution into a ptBLM using *in situ* dialysis. Tr-SEIRAS was performed in the step-scan mode under anaerobic conditions by applying periodic potential pulses stepping between −800 and +400 mV at a modulation frequency of 100 Hz [19].

**Figure 1:**
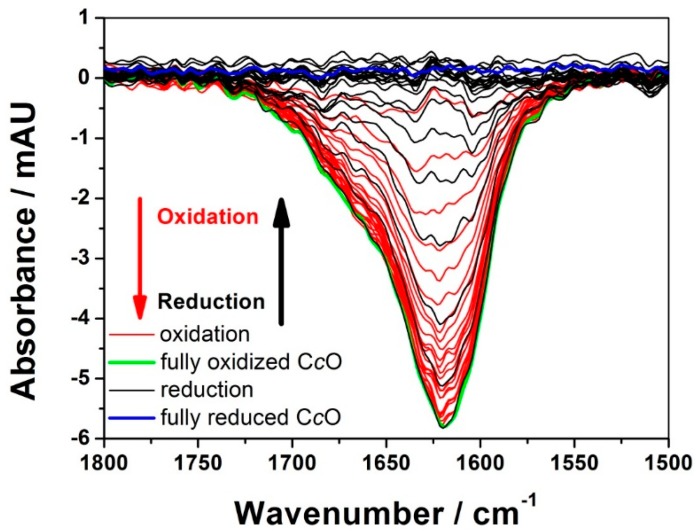
Time-resolved surface-enhanced infrared absorption spectroscopy (tr-SEIRAS) spectra of the activated cytochrome *c* oxidase (C*c*O) under anaerobic conditions. Spectra of the first excitation half period (oxidation) are shown in red, and those of the second half period (reduction) are shown in black. Spectra were taken over time intervals of 200 µs. Reproduced with permission from Schwaighofer *et al.* (2013) [19].

**Figure 2:**
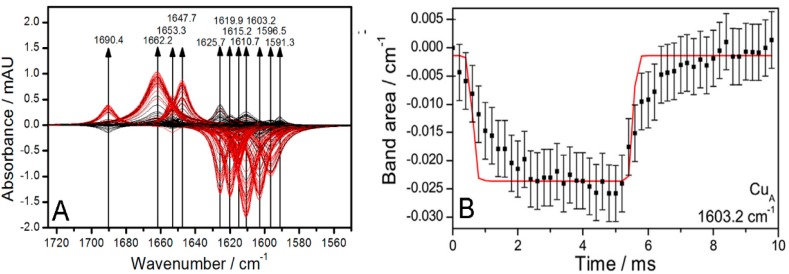

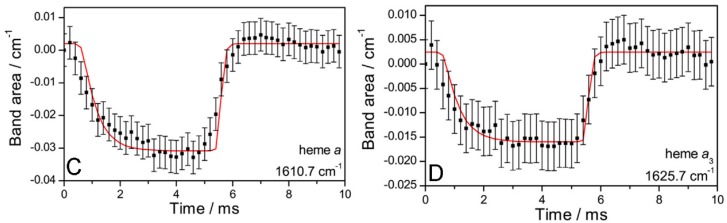
(**A**) Simulated spectra of deconvoluted bands revealed by phase sensitive detection (PSD) at an excitation frequency of 100 Hz. Subtraction of the OH bending band reveals positive and negative bands that are attributed to oxidized and reduced species, respectively. Spectra obtained in the first and second excitation half periods are shown in red and black, respectively; (**B**–**D**) Plots of band areas *vs.* time (black squares) of the bands at 1603.2 (Cu_A_), 1610.7 (heme *a*) and 1625.7 cm^−1^ (heme *a*_3_). The fitted curves of band areas *vs.* time to the sequential four-electron transfer model of cytochrome *c* oxidase (C*c*O) are given as solid red lines. Reproduced with permission from Schwaighofer *et al.* (2013) [19].

**Figure 3:**
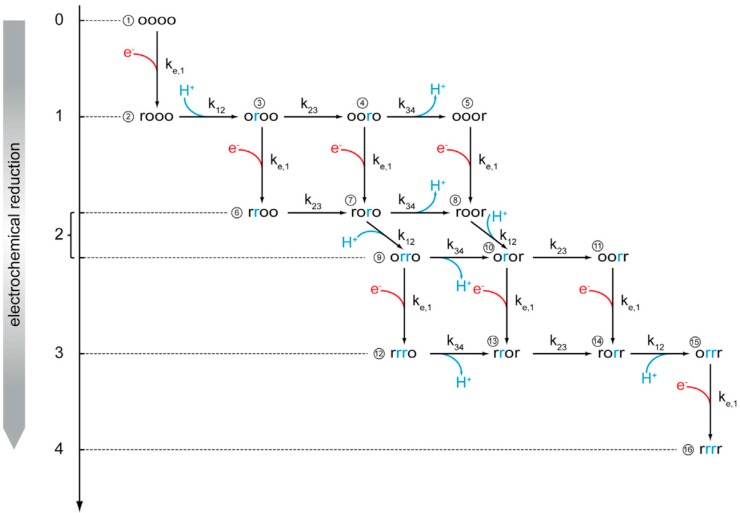
Kinetic scheme for sequential electron transfer in C*c*O under anaerobic conditions. The four centers are arranged in the order Cu_A_, heme *a*, heme *a*_3_, and Cu_B_, which can be present either in the reduced (r) or oxidized (o) state. All of the r and o states can be protonated or deprotonated. Hence, the enzyme can exist in 16 different redox states and 256 protonation states. The vertical arrows with red branches represent electron uptake from the electrode, whereas the black horizontal arrows indicate second-order reactions between redox centers. The blue arrows indicate proton uptake and release steps likely to occur as a consequence of the second-order ET reactions between redox centers. Reproduced with permission from Schwaighofer *et al.* (2013) [19].

As a result, a broad band was obtained in the region of the amide I bands (Figure 1), which, however, contained many overlapping bands, as revealed by a treatment called phase sensitive detection (PSD) (Figure 2A) [20]. PSD allowed us to subtract a large water band at 1643 cm^−1^ which otherwise would have obscured the bands of the secondary structures. The absorbance of this band was determined independently by measuring a SEIRA spectrum of a droplet of water on top of a Ni-NTA modified nano-structured gold surface. Details can be found in the supplementary information of reference [19]. Figure 2A shows PSD-deconvoluted tr-SEIRA spectra. The band areas obtained from Figure 2A were plotted as a function of time; see examples in Figure 2B–D used for the fitting routine, all of which are negative bands. The negative sign means they represent reduced species, which gradually disappear in the first half period of the potential pulse, going from −800 to +400 mV, and re-appear in the second half period, from +400 to −800 mV. The bands located at 1603, 1610 and 1625 cm^−1^, assigned to Cu_A_, heme *a*, and heme *a*_3_, respectively, were selected to be fitted to the kinetic model of the C*c*O, which is described below and in detail in reference [21,22]. See also Figure 3.

The same bands were found as in our titration study, although the distribution between the two types of secondary structures was quite different. In the titration, the helical structures are more prominent, while in the tr-SEIRA spectra the β-sheets are more strongly represented (see the bands at 1603, 1615, 1619, 1625 cm^−1^) while the band at 1653 cm^−1^, which is characteristic for α-helices, is weak in comparison. These findings agree well with the higher flexibility of β-sheets found in molecular dynamics (MD) studies of high-resolution crystallographic structures [23,24]. The greater rigidity of the α-helices, on the other hand, prevents these structures from following the excitation at high modulation frequencies, resulting in a lower absorbance in the respective IR-signals. Substantial conformational changes were also recently observed in separate high-resolution crystallography images of the reduced and oxidized form of C*c*O [25]. Bands at 1603.2, 1610.7 and 1625.7 cm^−1^ were assigned to changes in the redox state of Cu_A_, heme *a*, and heme *a*_3_, respectively.

The time-resolved absorbances of these bands were fitted to a kinetic model of proton transport through CcO (Figure 2). In this model, we use chemical reaction kinetics to explore proton transport coupled to electron transport (ET) in terms of a sequence of protonation-dependent second-order redox reactions, first presented in reference [21]. We thereby assume fixed rather than shifting dissociation constants of the redox sites. A schematic of the model is shown in Figure 3.

Bayesian inference was used to obtain the set of parameters that achieved the best agreement of experimental and simulated data. As fitting parameters, we chose the inherently pH-independent standard redox potentials and the pK values of the four redox centers in the order of sequential ET: Cu_A_, heme *a*, heme *a_3_*, and Cu_B_. As for the kinetic parameters, we must distinguish between electrochemical ET to Cu_A_, which is characterized by the electrochemical rate constant, and the chemical reaction kinetics between consecutive redox centers. Finally, τ, the time constant of charging the interface also has to be included. The kinetic constants of reactions between consecutive redox centers were found to be in the ms time scale and limited by electrochemical excitation. For further details, we refer to the original publication [19].

In this investigation, direct ET to a selected redox center of an MRP has thus been demonstrated to mimic the natural pathway of electrons through the enzyme. Electrochemical excitation has been shown as an alternative to the photolytic CO dissociation otherwise used to initiate enzyme turnover. The kinetics of enzyme reduction and oxidation, however, is limited by the ET rate to the electrode.

Later, we extended these studies to other multi-redox center proteins such as light-activated photosynthetic reaction centers from *Rhodobacter sphaeroides*.

#### 2.1.2. Photosynthetic Reaction Centers from *R. sphaeroides*

Reaction centers (RCs) with 7-his tags attached to the C-terminus of the M-subunit were reconstituted into a ptBLM as described. In this configuration, the special pair P/P+ is directed toward the gold film, whereas the Q_A_ and Q_B_ sites are directed toward the aqueous phase, and the ubiquinone pool is freely mobile within the lipid bilayer. As a further benefit, additional liposoluble Q_10_ can be co-reconstituted in controlled quantities. The formation of the entire structure was followed by electrochemical impedance (EIS) and SEIRA spectroscopy because the thin gold film can also be used as an electrode [26].

##### Light Activation of Reaction Centers (RCs) Followed by SEIRAS

**Figure 4:**
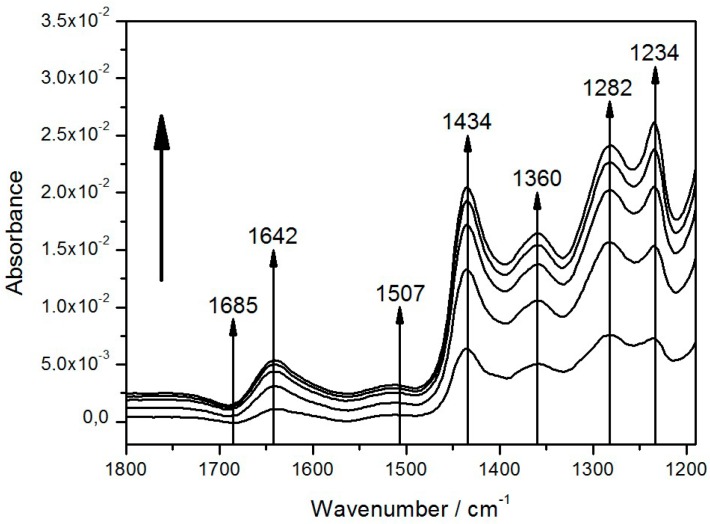
Light-minus-dark absorption spectra of reaction centers (RCs) encapsulated in the protein-tethered bilayer lipid membrane (ptBLM) taken at 5 min time intervals without added Q_10_ under continuous illumination. The term “light-minus-dark spectra” means that these are difference spectra using the spectra in the dark as references, which are subtracted from the spectra under illumination. The spectra were recorded every 5 min. Reproduced with permission of Nedelkovski *et al.* (2013) [26].

In this configuration, light-minus-dark SEIRA spectra were measured while the RCs were excited by continuous illumination under a halogen lamp first without any additional Q_10_. The term “light-minus-dark spectra” means that these are difference spectra that use spectra in the dark as references, which are subtracted from the spectra under illumination. A small number of prominent bands appeared, increasing over time in a time scale of minutes (Figure 4).

A steady state was not attained within a time scale of seconds as in previous investigations [27,28]. Nevertheless, the bands shown in our spectra could be correlated with characteristic groups found in previous studies. The tentative assignment of bands is shown in Table 1. The most prominent of these bands (1282 and 1434 cm^−1^) could be assigned to the oxidized form of the primary electron donor or special pair of the RC, P^+^, and ubihydroquinone, QH_2_, respectively, the products of light activation of the RCs. This was a surprising result, because in previous studies, appreciable amounts of QH_2_ could only be attained in the presence of an artificial electron donor, such as ascorbate P^+^, as it was reduced back to the reduced form of the special pair, P [29].

**Figure 5:**
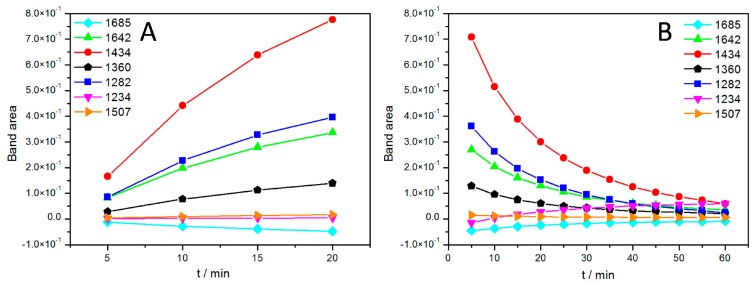
Band areas of the absorbances of characteristic bands of the RCs in the ptBLM under continuous illumination taken at different time intervals with added Q_10_ when the light is switched on (**A**) and off (**B**), *i.e.*, during relaxation. Reproduced with permission of Nedelkovski *et al.* (2013) [26].

The growth of the band areas was fully reversible, when the light was switched off, as shown in Figure 5B. Most band areas are reduced in the course of the relaxation with the exception of the bands at 1234 and 1685 cm^−1^, which are restored. This unexpected behavior combines several features characteristic for the SEIRA spectra of the ptBLM. Above all, the general pattern of the spectra is obviously different from the pattern of FTIR difference spectra usually displayed in the literature, which is mostly composed of narrow peaks and troughs [30].

The effect was even more pronounced when additional Q_10_ was co-reconstituted. The evolution of band amplitudes over time in the presence of additional Q_10_ is shown in Figure 5.

The reduced number of bands in the SEIRA spectra can be explained in terms of the surface selection rule, which comprises the high sensitivity of only those bands whose components are located at a short distance from the surface and the dependency of the sensitivity from the orientation of the transition dipole moments. The sensitivity is higher the more the component is oriented perpendicular to the surface [16,18]. According to this rule it depends on the orientation of a component whether it is strongly represented or not at all. This explains why the same functional group is represented by a certain band whereas other bands are missing. e.g., QH_2_ is usually represented by the bands 1434, 1491, 1470 cm^−1^, but only the band at 1434 cm^−1^ is definitively seen. This effect is amplified by the pre-orientation of the MRP molecules on the surface. Moreover, most of the bands appear to point in one direction, as seen in Figure 2A (C*c*O) and Figure 3 (RC). In the case of the C*c*O, this directionality can be explained by the superposition by the large water band. After subtraction, the bands point in both directions. In the case of the RC, all the bands are positive except for the 1685 cm^−1^ band, which is consistent with the fact that they represent products of the illumination. The only exception is the ground state of P, which is an educt. Another specific feature of the SEIRA spectra can be seen in the relatively large full width at half maximum (FWHM) of the bands, as compared to the sharp bands found in FTIR spectra of molecules present on the surface in a random orientation [30].The FWHM is associated with the freedom of movement of the particular structure represented by the component [32]. In previous studies, proteins were investigated mostly with redox mediators present. In our SEIRAS titration study of cytochrome *c* oxidase, we recorded a marked minimizing of the FWHMs in the presence of mediators [11,13]. This effect might be explained by the difference in internal dipole moments of the activated protein after the redox sites had been equilibrated with the mediators.

Irrespective of these general features of the SEIRA spectra, the slow evolution of species such as P^+^ and QH_2_, found in the case of the RC, is unexpected. In previous FTIR work, the release of QH_2_ from its binding pocket and its replacement by a quinone molecule from the quinone pool has been observed under the continuous illumination of chromatophores from *R. sphaeroides*, but only in the presence of ascorbate or other compounds to reduce P^+^ back to P [29]. The slow generation of species such as QH_2_ (and/or Q_B_H_2_) and P^+^, in the absence of an electron donor, can be explained by the reaction between two excited molecules of the RC.

P^+^Q_B_^−^ + P^+^Q_B_^−^ → P^+^Q_B_ + P^+^Q_B_^2−^ (1)

followed by protonating Q_B_^2−^, releasing QH_2_ into the membrane and then rebinding Q_B_ from the quinone pool. The model is supported by the fact that all of the light-induced SEIRA bands exhibit similar kinetics. Further support is derived from by the reversal of the original disproportion during relaxation (Figure 5B):

2P^+^Q_B_ + QH_2_ → P^+^Q_B_^2−^ + P^+^Q_B_ → P^+^Q_B_^−^ + P^+^Q_B_^−^ (2)

Thus, this study has demonstrated that inter-protein reactions are possible when the MRP is incorporated into the ptBLM. SEIRA spectra were again shown to be dominated by the surface selection rule, whose effects are strongly enhanced by the strict orientation of the protein perpendicular to the surface.

##### Direct Electron Transfer to Photosynthetic Reaction Centers from *R. sphaeroides*

The RCs were immobilized into a ptBLM as described above, with a genetically engineered 7-his tag at the C-terminus of the M-subunit such that the primary electron donor (the special pair P) was directed toward the gold electrode, in this case a smooth gold electrode. After illumination, P is converted into the oxidized species P^+^. Direct electron transfer (ET) to P^+^ was investigated, particularly in the context of generating photocurrents [33].

**Figure 6:**
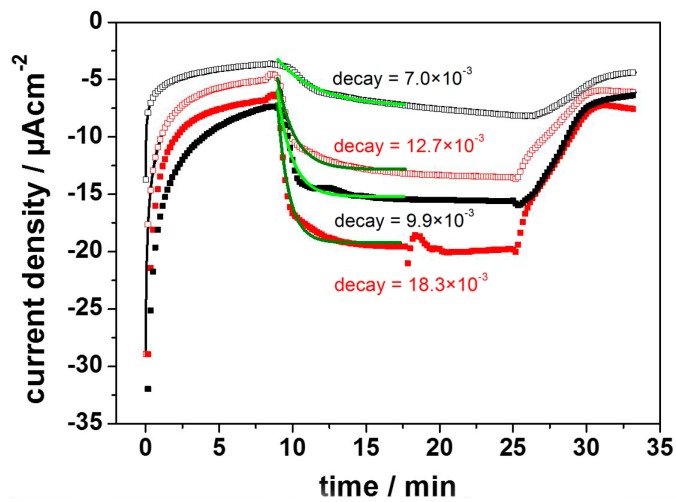
Photocurrents of RCs immobilized on a nitrilo-tri-acetic acid (NTA) modified template stripped gold (TSG) measured before (black) and after (red) formation of the ptBLM, at −200 mV (open squares) and −300 mV (full squares). Green lines are the monoexponential fits of the data. Reproduced with permission of Gebert *et al.* (2015) [33].

A very similar system had previously been introduced by the group of Lebedev and Trammel [34,35,36], who used linker molecules of different lengths to immobilize RCs on gold electrodes via his-tag technology, but without reconstitution into a ptBLM. Photocurrents were recorded in the range of nA after illumination of the RCs, but only when the photocurrents were mediated by cytochrome *c* (c*c*) and ubiquinone-2 (Q2). In our attempt to repeat these measurements in the presence of the BLM, we found dramatically different behavior. Substantial photocurrents lasting for more than 15 min in the range of µA were obtained in the cathodic direction in the range of negative potentials with an optimum at −200 mV. Photocurrents were even larger in the presence of the ptBLM (Figure 6) but not appreciably affected by the mediators Q2 and c*c*.

These comparatively high photocurrents were explained in terms of the disproportionation reaction (Equation (1)) discussed above. The direct electron transfer was attributed to the species P^+^Q_B_ rather than P^+^. This is consistent with the fact that we did not detect the photocurrents found by Trammel *et al.* at potentials of approximately 0 mV. We conclude that the photocurrents measured at −200 and −300 mV are due to direct ET to the species P^+^Q_B_. The corresponding oxidation peak is again between +600 and +650 mV, which can be explained in terms of the electrochemical reduction of P^+^Q_B_, which restores the ground state of the RC primary donor.

In summary, photocurrents of remarkable magnitude and stability have been generated, amplified by an inter-protein reaction in addition to the usual high energy conversion efficiency in RCs.

### 2.2. From Flat Surfaces to Particles

**Figure 7:**
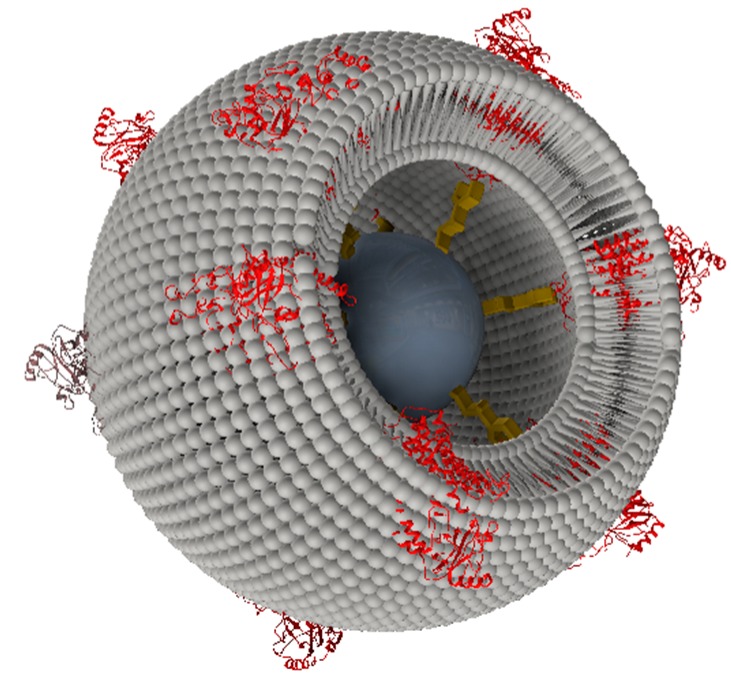
A schematic view of a proteo-lipobead (PLB). The agarose or silica bead is shown in dark grey, the NTA-terminated linker in yellow and the C*c*O in red, which is bound to the NTA-linker via a his-tag attached to the cytoplasmically oriented C-terminus of subunit I. The lipid bilayer is shown in light grey. The layered structure is not drawn to scale. Reproduced with permission of Frank *et al.* (2015) [37].

Flat surfaces are well designed for the application of electrochemical and surface-enhanced techniques such as surface plasmon resonance, surface plasmon-enhanced fluorescence and vibrational spectroscopies. Localized fluorescence effects, however, are not easily accessible on flat surfaces. Methods such as laser scanning confocal microscopy (LSM) are more suitable for 3D particles. Therefore, we transferred the ptBLM technique to commercially available particles of different sizes from µm down to nm, modified with NTA-terminated linkers. We referred to the obtained structures as proteo-lipobeads (PLBs) (Figure 7).

#### 2.2.1. Cytochrome *c* Oxidase from *P. denitrificans* Incorporated in Proteo-Lipobeads (PLBs) Based on Agarose Beads

C*c*O from *Paracoccus denitrificans* with a his-tag engineered to the C-terminus of the subunit I was bound to agarose beads (HisPur Ni-NTA Resin, 50–150 μm, Thermo Scientific, Schwerte, Germany), followed by *in situ* dialysis in the presence of lipid micelles to form PLBs [37].

**Figure 8:**
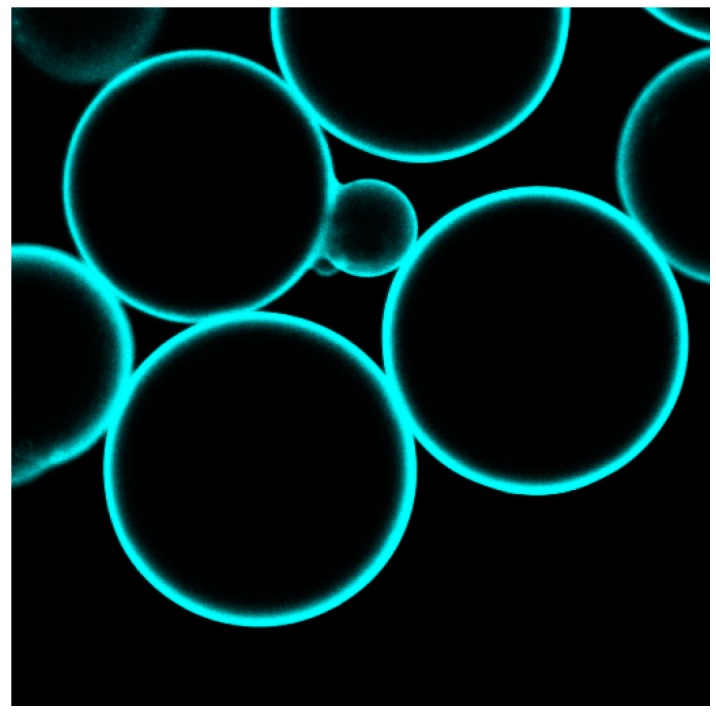
Confocal laser scanning microscopy (LSM) images of PLBs based on agarose beads. PLBs are labeled with the potential-sensitive dye di-4-ANBDQBS (4-(1-[2-(di-n-butylamino)-6-naphthyl]-4-butadienyl)-1-(4-butylsulfonate) quinolinium betaine). Reproduced with permission of Frank *et al.* (2015) [37].

The formation of the lipid layer was demonstrated by LSM, employing fluorescence-labeled lipids such as 1,2-dihexadecanoyl-sn-glycero-3-phospho-(*N*-4-nitrobenz-2-oxa-1,3-diazolyl)ethanolamine (NBD-PE) and the potential-sensitive dyes di-8-ANEPPS (di-8-butyl-amino-naphtyl-ethylene-pyridinium-propyl-sulfonate) and the red-shifted dye di-4-ANBDQBS (4-(1-[2-(di-n-butylamino)-6-naphthyl]-4-butadienyl)-1-(4-butylsulfonate) quinolinium betaine) (Figure 8). Hemicyanine-based membrane probes, such as di-8-ANEPPS and di-4-ANBDQBS, fluoresce strongly when bound to BLMs whereas they exhibit negligible fluorescence in aqueous solution [38]. Figure 8 illustrates a BLM layer with regular fluorescence intensity around the bead.

**Figure 9:**
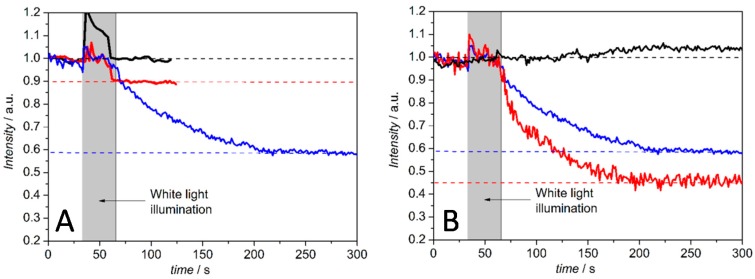
Change in the relative fluorescence intensity of membrane-bound pH-sensitive fluorescein DHPE ((1-(8-((3′,6′-dihydroxy-3-oxo-spiro(isobenzofuran-1(3H),9′-(9H)xanthen)-5-yl)amino)-3-hydroxy-8-thioxo-2,4-dioxa-7-aza-3-phosphaoct-1-yl)-1,2-ethanediyl ester, P-oxide) over time, before and after continuous illumination with a halogen lamp. (**A**) Comparison of unbuffered (40 mM KCl) (blue) and buffered (35 mM KCl, 5 mM Tris-HCl) (red) PLB solutions, each of them containing Ru_2_C ([(bpy)_2_Ru(diphen)Ru(bpy)_2_](PF_6_)_4_), 3CP (3-carboxy-PROXYL) and aniline to enable light excitation, and buffered PLB solution without the Ru2C, 3CP and aniline (black); (**B**) Change in the relative fluorescence intensity of pH-sensitive membrane bound fluorescein DHPE over time, before and after continuous illumination with a halogen lamp with Ru_2_C, 3CP and aniline in a 40 mM KCl solution (blue), in the presence of valinomycin alone (red) and with both valinomycin and FCCP (carbonyl cyanide 4-(trifluoromethoxy)phenylhydrazone) (black). The pH was adjusted to 8 before all measurements. Reproduced with permission of Frank *et al.* (2015) [37].

In the configuration above, the c*c* binding site of the C*c*O is pointing toward the outside of the PLB. Hence proton transport can be initiated by photoactive electron donors such as the Ru complex Ru_2_C ([(bpy)_2_Ru(diphen)Ru(bpy)_2_](PF_6_)_4_), which after light-activation acts as an electron donor, bound to the c*c* binding site. Ru_2_C was mixed with aniline as a sacrificial electron donor and 3CP (3-carboxy-PROXYL) to prevent proton release from the aniline [39]. The pH changes at the outer surface of the PLB were detected by fluorescein DHPE (1-(8-((3′,6′-dihydroxy-3-oxo-spiro(isobenzofuran-1(3*H*),9′-(9H)xanthen)-5-yl)amino)-3-hydroxy-8-thioxo-2,4-dioxa-7-aza-3-phosphaoct-1-yl)-1,2-ethanediyl ester, P-oxide), a sensor molecule that incorporates into the distal leaflet of the lipid bilayer. The decrease in fluorescence intensity indicates a decrease in pH in the outer solution. The fluorescence intensities were measured after continuous illumination with a halogen lamp with and without a mixture of Ru_2_C, 3CP and aniline. Fluorescence intensities decreased to an extent of 10% and 42% of the initial value in the buffered and unbuffered KCl solution, respectively. No decrease was observed in the presence of valinomycin and FCCP (carbonyl cyanide 4-(trifluoromethoxy)phenylhydrazone), *i.e.*, under uncoupling conditions. The intensity decreased to 55% of the initial value with only valinomycin present, which is explained by the collapse of the membrane potential (Figure 9). This behavior is in accordance with an active transporter controlled by the membrane potential.

#### 2.2.2. Cytochrome *c* Oxidase from *P. denitrificans* Incorporated in PLBs Based on Silica Nanoparticles

The size of the agarose beads (50–150 µm) described above, is very well suited for the application of LSM. However, they are not designed for the application of optical spectroscopy because they would suffer from serious interferences by light scattering of the particles. Therefore, we used commercially available 25 nm sized Ni-NTA-modified silica nanoparticles (NPs) to prepare PLBs designed for UV/Vis measurements [40].

**Figure 10:**
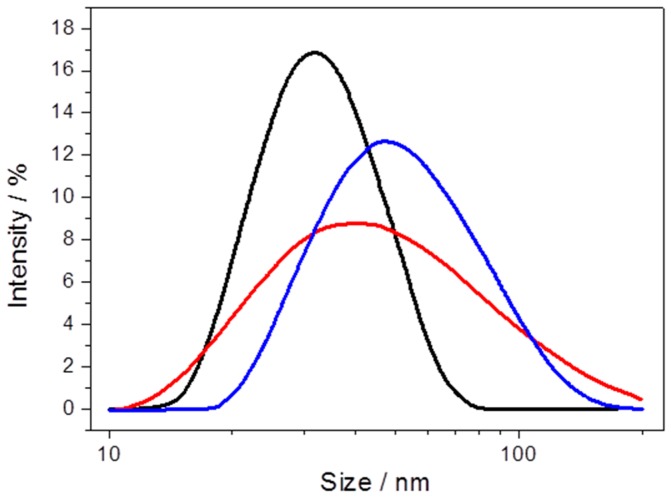
Particle size of PLBs, measured by dynamic light scattering of Ni-NTA-functionalized nanoparticles (NPs) (black), after C*c*O binding (red) and after dialysis (blue). Reproduced with permission of Schadauer *et al.* (2015) [40].

Particle size was measured by dynamic light scattering in the course of PLB preparation, starting with the NPs before and after C*c*O binding and after incorporation of the lipid bilayer. The size distribution increases regularly from 25 to 40 and 50 nm, for the NPs, the proteobeads and the PLBs, respectively (Figure 10). This increase is in accordance with crystallographic data for C*c*O, which predicts a height of 12 nm in the upright position [41].

**Figure 11:**
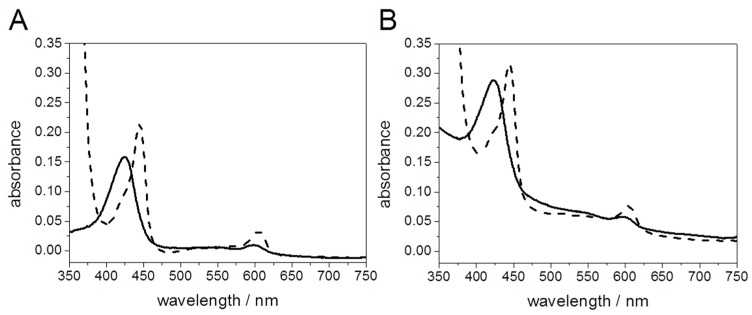
UV/Vis spectra of solubilized C*c*O (**A**) and PLB-bound C*c*O (**B**) oxidized with potassium ferricyanide (solid line) and reduced with sodium hydrosulfite (dashed line), respectively, at a C*c*O concentration of 1.1 µM. Reproduced with permission of Schadauer *et al.* (2015) [40].

UV/Vis spectra of C*c*O solubilized and C*c*O encapsulated in PLBs, before and after reduction with dithionite are shown in Figure 11. Light scattering effects are indicated by a sloping baseline, particularly at lower wavelengths. However, there is no interference of light scattering with the spectroscopic bands used for the determination of the concentrations of C*c*O, obtained using an extinction coefficient $\varepsilon_{606-630\;nm}^{reduced-oxidized}\;=$ 23.4 mM^−1^·cm^−1^ [42].

The activity of the incorporated C*c*O was assessed using a model based on the Michaelis-Menten equation. To this end, the reaction of the enzyme with reduced c*c* was followed, using solubilized C*c*O as well as the protein incorporated in proteobeads and PLBs. The measurements of the PLBs were also performed with 0.5 µM valinomycin and 1.2 µM FCCP present. The initial rates plotted against the c*c* concentration were fitted to the Michaelis-Menten equation. Fit parameters were the turnover number, *k*_cat_, and the substrate affinity, *K*_M_. The kinetic constants are collected in Table 2.

*k*_cat_ = 264 s^−1^ of solubilized C*c*O was found to reflect literature values [43]. It was found to decrease to a *k*_cat_ = 32 and 11 s^−1^ for proteobeads and PLBs, respectively. *k*_cat_ of C*c*O incorporated in PLBs recovered in the presence of valinomycin and FCCP to *k*_cat_ = 33 s^−1^. The reduction of *k*_cat_ = 32 s^−1^ was considered as being due to the immobilization of the proteins. The further reduction to *k*_cat_ = 11 s^−1^ within the PLBs was considered in terms of the lipid membrane. This conclusion seems justified in view of *k*_cat_ = 33 s^−1^ found under uncoupling conditions. From these values, the respiratory control ratio (RCR), was calculated to be 2.8. This is well in accordance with the RCR of 2.7 observed in proteo-liposomes [44]. The binding constant *K*_M_ = 5.8 µM of solubilized C*c*O was found to agree with literature data [43]. It decreased to 1.6 and 2.6 µM for proteobeads and PLBs, respectively. Summarizing, C*c*O within the PLBs suffers from a drastic decrease in activity, however, exhibiting improved binding properties.

## 3. Conclusions

These results demonstrate that the ptBLM structure is useful as a tool for biosensing. The main advantage of the ptBLM over traditional systems such as solubilized proteins or proteoliposomes is the strict orientation of the proteins embedded in the lipid bilayer. Other methods may be feasible in this context, such as electrostatic or antibody binding. Antibodies, however, usually have much larger dimensions compared with the combined his-tag and NTA-linkers. Hence, the proteins under investigation would be located outside the region of the evanescent wave of the EM field and thus would not be amenable to surface enhancement techniques such as SEIRAS and SERRS. Larger distances between the electrode and protein would also seriously hinder direct ET, which is relevant, for example, in the context of photocurrents. Electrostatic binding, on the other hand, is not as specific as his-tag binding. Moreover, purified proteins are now generally available genetically engineered with his-tags.

A method particularly well designed for use with ptBLMs is SEIRA spectroscopy. Electrochemical and light activation can be easily applied in a periodic fashion so that high quality S/N ratios can be easily obtained in tr-FTIR measurements. The surface selection rule, which works in favor of certain vibrations while others are silenced, presents a drawback. This feature, however, may actually be an advantage if the orientation of certain secondary structures is an aspect of interest. In any case, conformational changes in secondary structures with time can be observed, which is an important issue, particularly because time-dependent X-ray spectroscopy is not yet available.

The ptBLMs may also be considered for biosensing MRP inhibitors. They are frequently applied as herbicides because of their function as inhibitors, for example against complex III of the respiratory chain. There is high interest in biosensing these compounds with high sensitivity and selectivity.

The ptBLMs are not only significant in the context of biosensing but also in the context of basic research. We have strong evidence that under the conditions described here, the natural pathway of electrons all the way through the protein is maintained, particularly in the case of the C*c*O.

As for the methods to be applied, ptBLMs on flat surfaces allow the application of external electric fields and surface sensitive techniques such as surface plasmon resonance, surface plasmon-enhanced fluorescence and vibrational spectroscopies. PLBs based on spherical particles, on the other hand, allow for fluorescence microscopy and classical UV/Vis measurements. Fluorescence methods applied to PLBs open the way to bioassays for screening purposes including high-throughput screening. Hence, a whole range of methods is available to study MRPs under nearly physiological conditions.

## Figures and Tables

**Table 1:** Tentative band assignments of SEIRAS spectra of RCs encapsulated in the ptBLM recorded under continuous illumination [26]. Reproduced with permission of Nedelkovski *et al.* [26].

Band (cm^−1^)		Tentative Assignment	
Experimental	Literature	Species	Component
1282	1282	P^+^	(complex)
1360	1355, 1365	Q_A_	–CH_3_
1434	1433	QH_2_	
1643	1640, 1641, 1642 [31]	quinone Q, Q_B_	C=O
1685	1682, 1683	9-keto group of P	C=O
3400	3485	Q_B_^−^/Q_B_ or P^+^	H_2_O
3629	3632	Q_B_^−^/Q_B_ or P^+^	H_2_O

**Table 2:** Kinetic parameters derived from Michaelis-Menten kinetics for C*c*O incorporated in PLBs. Reproduced with permission of Schadauer *et al.* (2015) [40].

Parameter	C*c*O, Solubilized	C*c*O, in Proteobeads	C*c*O, in PLBs	
			−Val./FCCP	+Val./FCCP
*K*_M_ (µM)	5.8 ± 0.4	1.6 ± 0.4	2.6 ± 0.4	2.7 ± 0.6
*k*_cat_ (s^−1^)	264.1 ± 7.4	32.4 ± 0.5	11.9 ± 0.8	33.4 ± 8.8
Number of preparations		3	3	3
Number of measurements	3	3	3	3
